# Screening, identification, and characterization of molds for brewing rice wine: Scale-up production in a bioreactor

**DOI:** 10.1371/journal.pone.0300213

**Published:** 2024-07-02

**Authors:** HuaWei Yuan, Zhongyu Wu, HaoYu Liu, Xue He, ZhengWei Liao, WenJie Luo, Li Li, LiGuo Yin, Fang Wu, LiQiang Zhang, CaiHong Shen, SongTao Wang, JianLong Li, Tan Li, Kai Lou

**Affiliations:** 1 Faculty of Quality Management and Inspection & Quarantine/Solid-State Fermentation Resource Utilization Key Laboratory of Sichuan Province, Yibin University, Yibin, Sichuan, China; 2 School of Radiology, Shandong First Medical University, Jinan, Shandong, China; 3 Luzhou Laojiao Co., Ltd./Luzhou Pinchuang Technology Co., Ltd./National Engineering Technology Research Center of Solid-state Brewing, Luzhou, Sichuan, China; 4 College of Food Science, Sichuan Agricultural University, Ya’an, Sichuan, China; University of Patras, GREECE

## Abstract

Rice wine, well known for its unique flavor, rich nutritional value, and health benefits, has potential for extensive market development. *Rhizopus* and *Aspergillus* are among several microorganisms used in rice wine brewing and are crucial for determining rice wine quality. The strains were isolated via Rose Bengal and starch as a combined separation medium, followed by oenological property and sensory evaluation screening. The strain exhibiting the best performance can be screened using the traditional rice wine Qu. The strains YM-8, YM-10, and YM-16, which exhibited strong saccharification and fermentation performance along with good flavor and taste, were obtained from traditional rice wine Qu. Based on ITS genetic sequence analysis, the YM-8, YM-10, and YM-16 strains were identified as *Rhizopus microsporus*, *Rhizopus arrhizus*, and *Aspergillus oryzae*. The optimum growth temperature of each of the three strains was 30°C, 32°C, and 30°C, and the optimum initial pH was 6.0, 6.5, and 6.5, respectively. The activities of α-amylase, glucoamylase, and protease of YM-16 were highest at 220.23±1.88, 1,269.04±30.32, and 175.16±1.81 U/g, respectively. The amino acid content of rice wine fermented in a 20-L bioreactor with the three mold strains was higher than that of the control group, except for arginine, which was significantly lower than that of the control group. The total amino acid content and the total content of each type of amino acid were ranked as YM-16 > YM-8 > YM-10 > control group, and the amino acid content varied greatly among the strains. The control group had a higher content, whereas YM-8 and YM-16 had lower contents of volatile aroma components than the control group and had the basic flavor substances needed for rice wine, which is conducive to the formation of rice wine aroma. This selected strain, YM-16, has strong saccharification and fermentation ability, is a rich enzyme system, and improves the flavor of rice wine, thereby demonstrating its suitability as a production strain for brewing.

## 1. Introduction

Rice wine, a traditional alcoholic beverage of China, is renowned for its distinctive aroma, refreshed taste, and high nutritional value. Rice wine contains more than ten types of amino acids, eight of which are essential to the human body. Additionally, it has a lysine content several times higher than that of grape wine and beer. Rice wine contains glucose, maltose, and other small-molecule sugars that are readily metabolized by the body and yield more energy than beer and grape wine. Rice wine contains organic acids, minerals, and vitamins [1–4]. Studies have demonstrated the health benefits of rice wine, such as improvement in skin moisture, promotion of lactation, lowering blood pressure, and enhancement of immunity. However, the effects of moderate rice wine consumption on human health may vary among individuals. Additionally, rice wine has been found to modestly inhibit acetylcholinesterase activity, whereas aminobutyric acid has a potent inhibitory effect on angiotensin-converting enzymes [5–7]. These factors have contributed to the widespread popularity of rice wine.

Molds are vital for the saccharification of wine during fermentation. The flavor of rice wine can be improved by screening for fermentation molds with certain characteristics and systematically studying the fermentation performance and growth characteristics of the molds. Dung et al. [8] isolated and identified *Amylomyces rouxii*, *Amylomyces* aff. *rouxii*, *Rhizopus oligosporus*, and *Rhizopus oryzae* from rice wine, which degrade starch to produce glucose and starch glucosidase. Machida et al. [9] have successfully decoded the genome of *Aspergillus oryzae*. Abedinifar et al. [10] found that *Aspergillus oryzae* can produce many hydrolytic enzymes and flavor compounds, such as lactic acid, fumaric acid, alcohols, and esters, under certain cultivation conditions. Xiang et al. [11] co-cultured and fermented rice wine using *Mucor indicus* and *Rhizopus oryzae* and determined the flavor substances at different fermentation stages. The results showed that co-cultivation can significantly increase the type and content of flavor substances in rice wine, including esters, advanced alcohols, and organic acids, and significantly increase the content of sweet amino acids, making the aroma of rice wine stronger, and the taste fuller and more harmonious. Qu is a fermentation agent used in the brewing process and is produced by the spontaneous inoculation of rice or other grains containing a culture mixture of molds, yeasts, and bacteria, which are then cultured under suitable temperature and humidity conditions. During the cultivation process, molds produce various enzymes, particularly amylases, which are capable of breaking down starch in grains into sugars that can subsequently be converted by yeast into alcohol and carbon dioxide. There are various types of Qu depending on the material used to make them and the region; for example, those made from rice are called rice Qu, and those made from wheat are called wheat Qu [12]. However, screening for good-quality molds in rice Qu and brewing high-quality rice wine using rice as a raw material have not been sufficiently investigated.

In this study, the saccharification and fermentation abilities of rice wine fermentation molds were evaluated by measuring the alcohol content of rice wine. To obtain the most effective molds, it is necessary first to isolate and purify the different types of molds found in the traditional rice Qu. Subsequently, their oenological properties should be analyzed in conjunction with the sensory evaluation of rice wine. This comprehensive approach will help identify the molds that exhibit the highest performance. This mold imparts a pleasant and unique taste to fermented rice wine and has practical implications for maintaining or improving its flavor and quality of rice wine.

## 2. Materials and methods

### 2.1 Isolation of molds from traditional Qu

The ferment starter Qu, used in brewing traditional rice wine, was obtained from different regions of China, namely, Dazhou, Yibin, Xiaogan, and Laibin.

A 5-g sample of Qu was weighed and added to 50 mL of potato dextrose agar (PDA) in liquid medium (glucose, 20 g; peeled potato, 200 g; distilled water, 1,000 mL; pH 7.0; sterilized at 121°C for 15 min) and cultured at 28°C and 100 r/min for 48 h in a thermostatic incubator (model ZWY-211C, Shanghai Zhicheng Analytical Instrument Factory, Shanghai, China). A 2-mL volume of the culture was inoculated on bran medium (wheat bran, 50 g; water, 40 g; sterilized at 121°C for 15 min) and cultured in an incubator at 28°C. The mixture was shaken once after 18 h of the initial culture and then shaken again after 24 h. Culture was completed after 48 h, at which time many spores were formed. Sterilized water (100 mL) was added, the mycelia were scattered with sterilized glass beads, and the spore suspension was obtained by filtering through sterilized double-layer gauze [13, 14].

The spore suspension was diluted in a gradient, and 0.1 mL of suitable diluted spore suspension was collected by suction and coated on Rose Bengal medium (glucose, 10 g; peptone, 5 g; agar, 15 g; KH_2_PO_4_, 1 g; MgSO_4_, 0.5 g; Bengal red, 0.03 g; chloramphenicol, 1.0 g; distilled water, 1,000 mL; pH 7.0; sterilized at 121°C for 15 min) and cultured at 28°C. Single colonies with detectable mold morphology were selected and inoculated onto a PDA slope (15 g/1,000 mL agar) for preservation [15].

The selected mold strains were cultured on starch medium (soluble starch, 2 g; peptone, 10 g; beef extra, 5 g; NaCl, 5 g; agar, 15 g; distilled water, 1,000 mL; pH 7.0~7.2; sterilized at 121°C for 15 min) at 28°C for 24 h, and then 3 mL of Lugol’s iodine solution was dripped onto them. The ratio of the diameter of the clear zone to that of the colony was measured after the transparent circles became evident. Strains with larger ratios were selected for rice-wine fermentation testing [16].

### 2.2 Rice wine fermentation experiments

**The** *Saccharomyces cerevisiae* utilized in this study originated from the traditional rice wine starter Qu and was preserved in the solid-state fermentation resources utilization key laboratory of Sichuan province. Yeast was inoculated on yeast peptone dextrose (YPD) medium (yeast extract, 10 g; peptone, 20 g; dextrose, 20 g; sterilized at 121°C for 15 min) and cultured at 25°C for 72 h. Yeast cultures containing no fewer than 3 × 10^8^ cells/mL were collected, as determined by direct counting in a hemocytometer chamber [17].

The screened mold strains were applied to bran medium to formulate a spore suspension for preparing rice *koji*. The rice *koji* culture was prepared in an incubator (model HMJ-II-300, Shanghai Yuejin Medical Instrument Co., Ltd., Shanghai, China) at a certain temperature and humidity according to a previously reported method [18].

An 82.5-g sample of rice *koji* was placed in a 1,000 mL jar, to which 100 mL of sterilized water and 1 mL of yeast culture were added. The first fermentation was performed at 25°C for 3 d. A 250-g sample of rice was cleaned, soaked, cooked, cooled to 35°C, and transferred to a culture jar, to which 450 mL of sterilized water was added. The secondary fermentation was performed at 25°C for 13 d. The fermentation mash was shaken manually once daily, and rice wine was obtained after filtration, according to a previously described method [19]. Rice wine fermented by traditional Qu was used as a control. The rice wine was stored at 4°C for further analysis. All procedures were performed in triplicate. The enological parameters, alcohol content, and sensory characteristics of the rice wine were evaluated. The selected molds were used for further identification and characterization.

The ethanol, total sugar, total acid, pH, and amino nitrogen content of the rice wine were analyzed following the GB/T 13662-2018 method (China) [20]. Sensory evaluation of the rice wine was conducted according to a previously reported method, and scores were obtained by well-trained panelists [19]. A total score of 10 indicated excellent wine quality.

### 2.3 Molecular taxonomy of the selected molds

The selected isolates YM-8, YM-10, and YM-16 were cultured in PDA medium inoculated with a mold spore suspension at 28°C for 48 h. The thalli were collected by centrifuging the cultures at 10,000 × g for 5 minutes after transferring the cultures to centrifuge tubes. The Universal DNA Purification Kit (TiangenTM, Beijing, China) was used to extract genomic DNA from the recovered thallus. Using the primers ITS1 (5’-TCCGTAGGTGAACCTGCGG-3’) and ITS2 (5’-TCCTCCGCTTATTGATATGC-3’), the ITS domain of the 5.8S rDNA region was amplified. The following conditions were used for PCR amplification: initial denaturation at 98°C for 3 min, then 39 cycles of 98°C for 10 s, 53°C for 10 s, and 72°C for 10 s/kb, and then a final extension at 72°C for 5 min. The Illumina MiSeq platform was used to sequence the amplified 5.8S rDNA ITS region. The obtained sequence from the chosen isolate was compared to GenBank. MEGA X was used to examine the acquired sequences’ phylogeny. The maximum composite likelihood evolutionary model was used to calculate distances on a phylogenetic tree, which was then bootstrapped with 1,000 replications. [21–23].

### 2.4 Growth characteristics and enzyme production ability of the selected molds

The influence of temperature and pH on the selected mold isolates was investigated in growth trials. The tested variables were temperature (24, 26, 28, 30, 32, 34, and 36°C) and initial pH (5.5, 6, 6.5, 7, 7.5, and 8). A 100-mL sample of liquid PDA medium was inoculated with 1 mL mold spore suspension and then cultured at 100 rpm for 36 h in a thermostatic incubator (model ZWY-211C, Shanghai Zhicheng Analytical Instrument Factory, Shanghai, China). The mycelium was filtered and dried in an oven at 110°C to constant weight, and the dry weight of mycelium was measured. The optimal growth temperature and pH were determined based on the dry weight of the mycelia.

The isolates were cultured continuously at the optimal growth temperature and pH, as described above, and the dry weight of the mycelium was measured every 3 h to draw the growth curve of the selected molds.

The activities of α-amylase, glucoamylase, and protease were determined using rice *koji* produced by the selected isolates, according to the QB/T1803-1993 method (China) [24]. The activity units were defined the following method. The unit activity of α-amylase (1 U/g), using 1 g rice *koji*, was defined as the liquefaction of 1 g of soluble starch in 1 h at 60°C and pH 6.0. The unit activity of glucoamylase (1 U/g) using 1 g rice *koji* was defined as the decomposition of 1 mg soluble starch in 1 h at 35°C and pH 4.6. The unit activity of protease (1 U/g), using 1 g rice *koji*, was defined as 1 μg tyrosine hydrolyzing casein in 1 min at 40°C and pH 3.0.

### 2.5 Use of the selected isolates to scale up rice wine production in a 20-L bioreactor

The scale-up process in a 20-L bioreactor was performed according to a previously published method [19]. A 1650 g sample of rice *koji* was introduced into a 20 L bioreactor, followed by the addition of 2000 mL of sterilized water and 20 mL of yeast culture. Subsequently, a 5000 g sample of rice was cleaned, soaked, cooked, cooled, and transferred to the same 20 L bioreactor, along with the addition of 9000 mL of sterilized water. The initial fermentation took place at a temperature of 25°C for three days. This was followed by a secondary fermentation at the same temperature for 13 days, during which the mixture was shaken daily. After filtration, the resulting product was rice wine. Rice wine was prepared using the selected mold strains and traditional Qu as a control, and its amino acids and volatile flavor compounds were measured.

Using high-performance liquid chromatography (HPLC) (model 1100, Agilent Technology Inc., Calif., USA) and an Agilent C18 column (250 mm 4.6 mm 5 m), the amino acid content of the rice wines was determined with some slight modifications to the early approach [25–27]. Mobile phase A (pH 7.2) contained 27.6 mmol/L sodium acetate, riethylamine, and tetrahydrofuran (500:0.11:2.5); mobile phase B (pH 7.2) contained 80.9 mmol/L sodium acetate, methanol, and acetonitrile (1:2:2); protocol for elution: 0 min., 8% B, 17 min., 50% B, 20.1 min., 100% B, and 24 min., 0% B; flow rate of 1 mL/min and a 40°C column temperature UV detector (VWD) detection wavelength: 338 nm; proline detection wavelength: 262 nm.

In accordance with a previously established approach with minor modifications [28–30], the volatile taste components of rice wine were examined using solid-phase microextraction (SPME) and gas chromatography–mass spectrometry (GC–MS). Three 3-mL samples of rice wine were placed in a 15-mL headspace bottle along with 1.2 g of NaCl and 10 L of the internal standard, 2-octanol (54.827 mg/L in absolute ethanol). An SPME device (Supelco Inc., Bellefonte, Pennsylvania, USA) with 50/30 m (DVB/CAR/PDMS)-coated fibers was employed. The rice wine samples were equally submerged in a continuous water bath, balanced at 60°C for 15 minutes, and then recovered for 45 minutes. The fiber was then injected into the GC injection port for 5 minutes at 250°C to desorb the analytes. It was used using an HP-INNOWAX column (60 m 0.25 mm 0.25 m, Agilent Technologies, Inc., CA, USA) and GC‒MS (QP2020, Shimadzu Co. Ltd, Kyoto, Japan). The temperature of the gas chromatography oven was raised from 40°C to 100°C and then from 6°C to 230°C over the course of five minutes, after which it was maintained for ten minutes. The carrier gas, helium, was employed and flowed at a rate of 1.0 mL/min (99.999%). The electron impact (EI) mode mass detector was employed at 70 eV with an ion source temperature of 230°C. The volatile flavor compounds were identified by contrasting the mass spectral database (NIST14s) and quantified by an internal standard approach according to a previous study [31].

### 2.6 Statistical analysis

Data from triplicate samples are reported as the mean values with standard deviation (SD) for enological parameters and enzyme activity. Using Duncan’s test at p < 0.05, significant differences among the data were identified. By using Origin 2018, free amino acid and taste component results were displayed. PLSR and Unscrambler (version 9.7, CAMO ASA, Oslo, Norway) were used to examine the potential connections between free amino acids, flavoring ingredients, and specific molds.

## 3 Results and discussion

### 3.1 Isolation and selection of mold strains

Ninety-six single colonies with mold morphology were selected for preliminary screening. The ability to hydrolyze starch was evaluated in mold strains isolated from traditional Qu used for brewing rice wine using a starch medium. In Table 1, we list the origins of the 10 mold isolates, diameter of the clear zone and colony, and ratios > 1.50. The results showed that the seven selected isolates produced sufficient ratios between 2.05±0.03 and 2.72±0.08. The best results were achieved with strains YM-13 (2.72±0.08), YM-10 (2.27±0.64), YM-4 (2.23±0.01), and YM-16 (2.21±0.06). Strains with higher ratios exhibit strong starch hydrolysis abilities [32]. The five isolates with the highest ratios were selected for rice-wine fermentation and sensory evaluation.

**Table 1:** Diameter and ratio of screening strains by plate.

Strains	Clear zone (mm)	Colony (mm)	Ratio (Clear zone/Colony)
YM-4	20.3±0.9	9.1±0.4	2.23±0.01
YM-5	20±0.6	11.5±0.6	1.74±0.03
YM-8	11.1±0.7	5.2±0.5	2.13±0.06
YM-10	13.6±0.2	6±0.8	2.27±0.64
YM-13	19.6±1.7	7.2±0.8	2.72±0.08
YM-16	21±0.7	9.5±0.6	2.21±0.06
YM-17	22.9±1.8	11.1±1.2	2.06±0.07
YM-18	12.9±0.7	7.2±0.8	1.79±0.09
YM-20	21.4±0.7	11.2±1.0	1.91±0.12
YM-26	18.7±0.9	9.1±0.4	2.05±0.03

The brewing characteristics of rice wine - ethanol content, total sugar, total acid, pH value, and amino acid nitrogen - as well as sensory evaluation scores are shown in Table 2. Overall, all evaluated brewing characteristics are within the scope specified in Chinese national regulations (GB/T13662-2018 "Chinese Yellow Wine").

**Table 2:** Enological parameters and sensory evaluation of rice wine brewing by selected strains.

Parameter	Control	YM-4	YM-8	YM-10	YM-13	YM-16
Ethanol (%, v/v)	13.82±0.23^a^	14.04±0.12^a^	10.50±0.12^c^	9.57±0.10^d^	12.49±0.16^b^	13.79±0.15^a^
Total sugar (g/L)	14.84±0.20^d^	9.51±0.08^e^	25.44±0.72^b^	33.28±0.82^a^	15.76±0.16^d^	18.42±0.84^c^
Total acid (g/L)	3.13±0.03^ab^	2.91±0.04^c^	3.10±0.03^b^	3.19±0.05^a^	2.88±0.04^c^	3.06±0.06^b^
pH	3.55±0.02^f^	3.78±0.01^c^	3.96±0.01^a^	3.61±0.02^e^	3.82±0.01^b^	3.65±0.02^d^
Amino-nitrogen (g/L)	0.35±0.01^e^	0.40±0.02^bc^	0.44±0.01^a^	0.39±0.01^cd^	0.38±0.01^d^	0.42±0.01^ab^
Total score	9.51±0.11^a^	7.51±0.17^c^	8.92±0.18^b^	9.14±0.28^b^	6.58±0.17^d^	9.42±0.16^a^

All values are means of triplicate mean values ± SDs. Means within different letters were significantly different (p < 0.05) on the same line.

The results showed that five isolates produced ethanol contents between 9.57±0.10% and 14.04±0.12%. The rice wine fermented by YM-4, YM-16, and YM-13 had a higher ethanol content, indicating a relatively greater ability of these three strains to hydrolyze starch. Rice wine fermented by YM-4 had the highest ethanol content, at 14.04±0.12%; however, the total sugar content was lowest, possibly due to the strong activity of amylase and glucoamylase derived from YM-4. Conversely, the rice wine fermented by YM-10 yielded the lowest ethanol content, 9.57±0.10%, but the highest total sugar content, at 25.44±0.72 g/L. The ethanol concentration from strain YM-4 was only slightly higher than that of the control group. The amino-nitrogen content in rice wine brewed with the selected molds was significantly different (p < 0.05). In the case of the YM-4, YM-8, and YM-16 treatment groups, the amino-nitrogen content of the fermented rice wine was significantly higher in the other treatment groups, likely due to differing levels of protease activity among the different strains [33].

The ability of molds to ferment rice is closely related to the flavor substances and ethanol content of rice wine; therefore, these factors should be considered in combination with sensory aspects for a comprehensive evaluation. The strains that yielded the highest rice wine scores were YM-16, YM-10, and YM-8. Rice wines fermented by the YM-16 strain and the control had the best flavor and highest score, followed by the YM-10 strain. The difference in the scores between the two strains was not significant. Rice wines fermented using YM-10 and YM-16 displayed conspicuous differences in taste. YM-10 had a light taste and lacked mellow and sour notes. The increased sweetness but lack of flavor in rice wines may be attributed to the insufficiently comprehensive characteristics of the enzyme system produced by a single strain during fermentation. Following a comprehensive evaluation, the YM-8, YM-10, and YM-16 strains were identified as ideal strains for brewing rice wine.

### 3.2 Identification of selected mold strains: Molecular characterization and taxonomy

**Fig 1:**
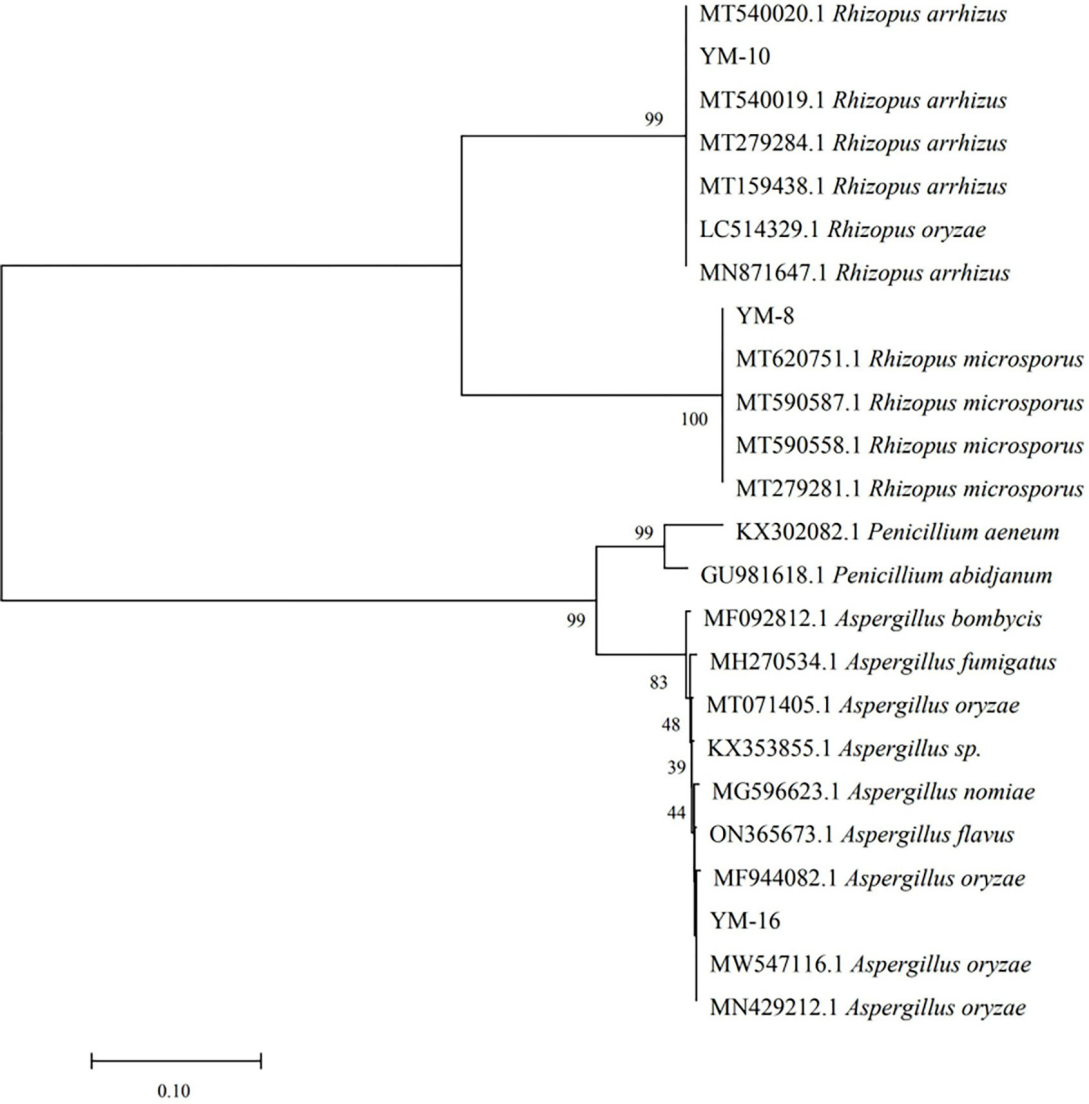
Phylogenetic tree of the screened molds.

The sequencing results from the NCBI database (obtained using BLASTN alignment of recognized standard sequence data of relevant species obtained from the GenBank database), alignment results (obtained using ClustalX 1.8 and MEGA 4 calculations of sequence similarity), and phylogenetic analysis are shown in Fig 1.

**Fig 2:**
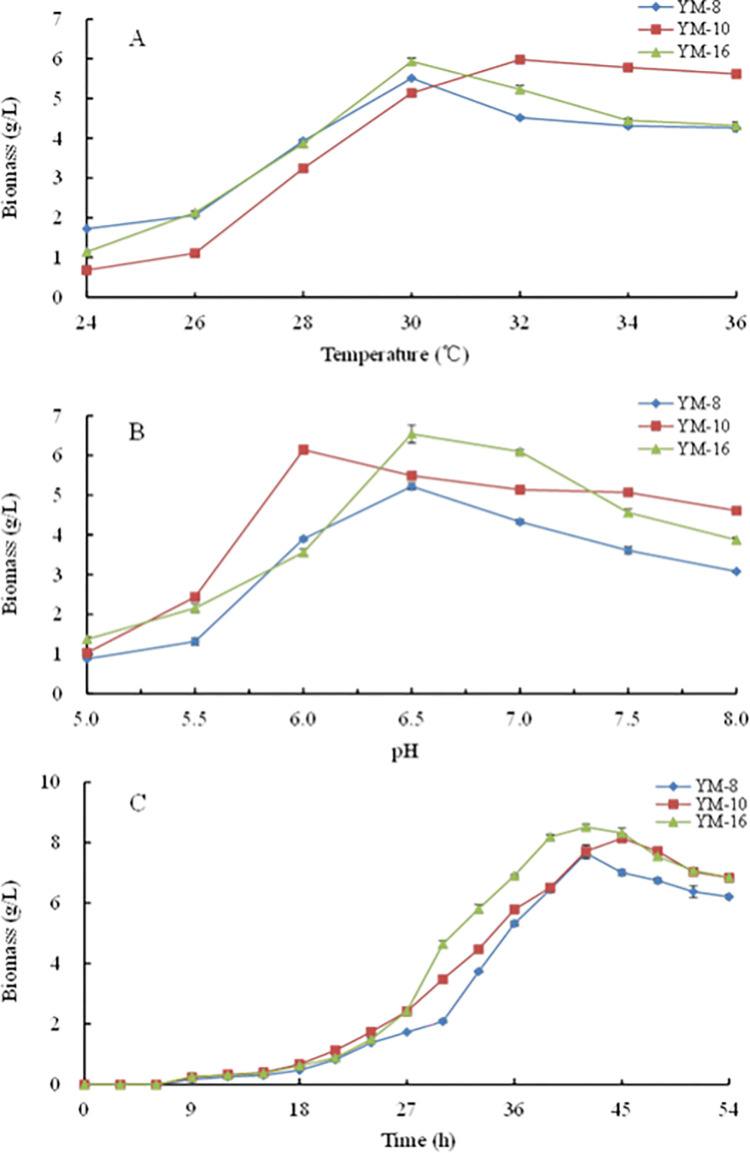
The effect of incubation temperature, pH, and time on the growth of screened strains.

Alignment of the rDNA sequences of these isolates showed that strain YM-8 has a degree of similarity of 100% with *Rhizopus microsporus* strain MT 620751.1. Strain YM-10 showed 100% similarity to *Rhizopus arrhizus* strain MT 5400201.1. Strain YM-16 was shown to have 100% similarity to *Aspergillus oryzae* strain MW 547116.1. To confirm the position of each strain in the phylogeny, several sequences were selected from the NCBI database to construct a phylogenetic tree using the MEGA 4 program. As shown in Fig 2, YM-8 and YM-10 belong to the genus *Rhizopus* and YM-16 belongs to the genus *Aspergillus*. The internal transcribed spacer (ITS) sequences of strains YM-8, YM-10, and YM-16 were compared using NCBI and identified as *Rhizoctonia microsporus*, *Rhizopus arrhizus*, and *Aspergillus oryzae*, respectively.

Comparing these results with previous studies, they are consistent with the findings that *Rhizopus* species are commonly associated with fermented food products, including soybean curd [34, 35]. Similarly, *Aspergillus oryzae* is a well-known fungus used in the fermentation process of various foods, including soybean products [36–38].

However, further investigation is needed to understand the specific roles and contributions of these strains in the fermentation process and their potential impact on the quality of the final product.

### 3.3 Growth characteristics of the selected mold strains

As shown in Fig 2A, at pH 6.5 for 36 h, the dry weights of the mycelia of YM-8 and YM-16 reached maximum values of 5.51±0.03 and 5.94±0.08 g/L, respectively. At 32°C, the dry weight of the mycelium of YM-10 reached the maximum value, at 5.98±0.10 g/L, whereas the dry weight of the mycelium of YM-8 and YM-16 decreased. Therefore, the optimal growth temperature was 30°C for YM-8 and YM-16 and 32°C for YM-10.

As shown in Fig 2B, at 30°C for 36 h, the optimal pH for the growth of YM-10 was 6.0, yielding a maximum mycelial dry weight of 6.14±0.11 g/L. The optimal pH for the growth of YM-8 and YM-16 was 6.5, with maximum mycelial dry weights of 5.22±0.08 and 6.54±0.22 g/L, respectively. The optimal initial pH values for the growth of YM-8, YM-10, and YM-16 were 6.5, 6.0, and 6.5, respectively.

At pH 6.5 for 30°C, the dry weights of the mycelium of YM-8 and YM-16 reached a maximum at 42 h, yielding 7.65±0.19 and 8.52±0.09 g/L, respectively, and the dry weight of mycelium of YM-10 reached a maximum at 45 h, yielding 8.14±0.10 g/L (Fig 2C). An incubation time of 40–47 h has been reported to be optimal for mold growth, with the strongest metabolic activity and production of primary metabolites enhancing rice wine quality [39].

### 3.4 Enzyme production characteristics of the selected mold strains

There were significant differences in the enzyme-producing capacity of different molds (Table 3), with glucoamylase having the highest activity, followed by α-amylase, and protease having the lowest activity. Fermentation of rice wine by molds is a simultaneous process of saccharification and fermentation: saccharification enzymes are secreted, and the saccharides produced by saccharification are available to yeast for alcoholic fermentation.

**Table 3:** Enzyme activity of screened strains.

Enzyme activity (U/g)	Control	YM-8	YM-10	YM-16
α-Amylase	209.50±2.37^b^	192.49±2.77^d^	204.73±1.65^c^	220.23±1.88^a^
Glucoamylase	938.67±22.23^d^	1146.42±25.95^b^	1084.51±15.71^c^	1269.04±30.32^a^
Protease	138.68±3.59^d^	165.88±5.50^b^	150.26±3.57^c^	175.16±1.81^a^

Protease hydrolyzes proteins, forming amino acids and peptides that provide nutrition and flavor for rice wine, so the richer the enzyme system of molds and the higher the enzyme activity, the more conducive to the formation of a wide variety of easily digestible small molecules of nutrients and aroma components, which contribute to the flavor of rice wine and yield carbon and nitrogen necessary for yeast growth and reproduction [33]. The levels of activity of glucoamylase, α-amylases, and protease were highest in strain YM-16, at 1,269.04±30.32, 220.23±1.88, and 175.16±1.81 U/g, respectively (Table 3). In a study by Yang et al. [40], the fermentation capability and quality of rice wine were significantly enhanced using a combination of fungal strains, including *Rhizopus chinonsis* R01, *Aspergillus niger* A20, *Mucor pusillus* M05, and *Saccharomyces cerevisiae* S10, as starters for japonica rice fermentation. This process was supplemented with α-amylase (AM), glucoamylase (GAM), and acid protease (AP). AM and GAM effectively broke down rice starch, reduced viscosity, and increased the enzyme contact area, which accelerated the hydrolysis of japonica rice. Furthermore, AP boosted the protease activity of *M*. *pusillus* M05 and *A*. *niger* A20, leading to improved protein utilization, increased free amino acid content, and the formation of higher levels of flavor substances in rice wine. These findings indicate that hydrolases can alter the physical and chemical properties of rice mixtures, thereby enhancing microbial biotransformation and modulating flavor metabolism during fermentation. Although our research focused solely on individual mold isolates, it aligns with previous studies, suggesting that specific strains of *Aspergillus oryzae*, such as YM-16, possess inherent enzymatic capabilities that contribute to the overall quality of rice wine. Forty-seven strains of *Aspergillus oryzae* were isolated from Korean nuruks, and their brewing characteristics were compared [41]. Among these strains, *A*. *oryzae* YI-A6 and YI-A7 showed the highest acid α-amylase, glucoamylase, and carboxypeptidase activities, respectively. Son et al. [42] investigated the effects of *Koji* inoculated with *Saccharomycopsis fibuligera* and *Aspergillus oryzae* on the volatile and nonvolatile metabolite profiles of *makgeolli*, a traditional Korean rice wine. They found that the protease activity of *S*. *fibuligera* CN2601-09 led to the highest fusel alcohol and acetate ester content, emphasizing the influence of microbial strains with different enzyme activities on the formation of metabolites in rice wine. These findings are consistent with the current research, as they suggest that certain strains of *A*. *oryzae* have higher enzymatic activity levels than others.

Overall, the three selected mold strains showed higher levels of enzyme activity than the corresponding values in the control group, indicating that the screened molds were suitable for rice wine nutrition and flavor formation. Further research should investigate the specific mechanisms underlying these enzymatic activities and explore the potential of mixed culture fermentation to further improve the quality of rice wine.

### 3.5 Use of selected molds to scale up rice wine fermentation in a 20-L bioreactor

#### 3.5.1 Free amino acid content of rice wine

**Fig 3:**
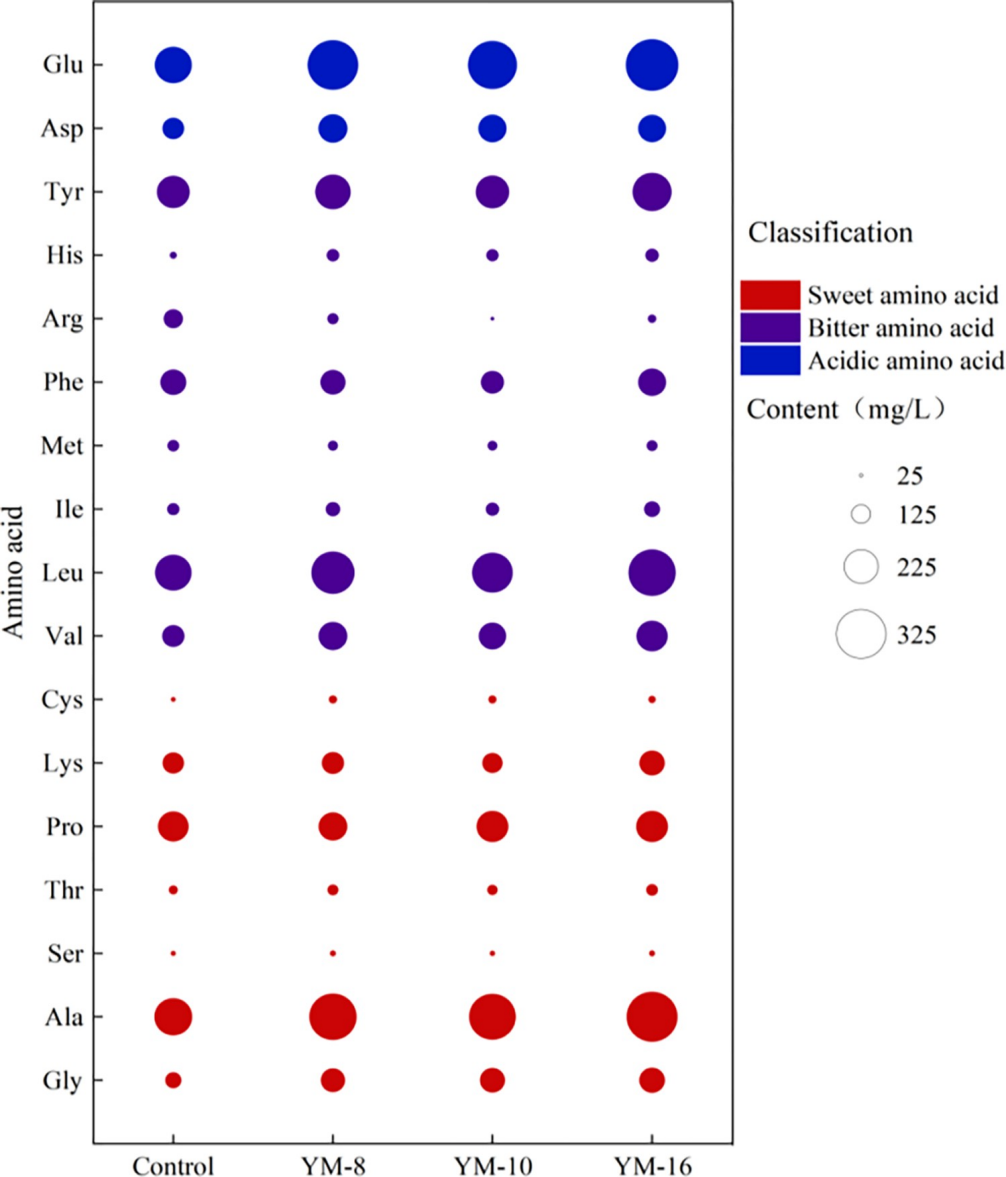
Enrichment analysis of free amino acids in rice wine using traditional Qu as a control.

Seventeen amino acids were detected in rice wine, which were mainly formed by protein enzymolysis and yeast autolysis. These amino acids can be categorized into sweet, bitter, and acidic amino acids [42, 43]. As shown in Fig 3, the amino acid content of rice wine fermented with the three mold strains was higher than that of the control group, except for arginine, which was significantly lower than that of the control group. The total amino acid content and the total content of each type of amino acid were ranked as YM-16 > YM-8 > YM-10 > control group, and the amino acid content varied greatly among the strains. These findings are consistent with previous studies on the amino acid composition of rice wines. Liang et al. [44, 45] investigated the dynamics of fungal and bacterial communities during the fermentation of Hong Qu glutinous rice wine and found that the amino acid content increased continuously under different temperature conditions. Gao et al. [46] analyzed the influence of key techniques on the composition, content, and taste characteristics of amino acids in Hakka rice wines from Guangdong Heyuan. They found that the total amino acid content of the rice wines ranged from 2658.99 mg/L to 5420.63 mg/L, with black soybean wine having the highest content and chestnut rice wine having the lowest. Glutamic and aspartic acids were the main amino acids in all rice wines, whereas cysteine, methionine, and phenylalanine were the limiting amino acids. Inoue et al. [47] characterized the physicochemical properties of Japanese rice wines, including their carbohydrate and amino acid contents, viscosity, and antioxidant capacity. They found that the glucose, allose, and raffinose contents varied across the samples, as did the total amino acid content and levels of specific amino acids such as glutamic acid, alanine, and arginine. Overall, these studies highlight the importance of studying the amino acid content of rice wines fermented with different mold strains. The variation in amino acid content among different rice wines and the influence of factors such as temperature, raw materials, fermentation process, brewing method, and storage time suggest that optimizing these parameters can improve the taste and quality of the final product.

**Fig 4:**
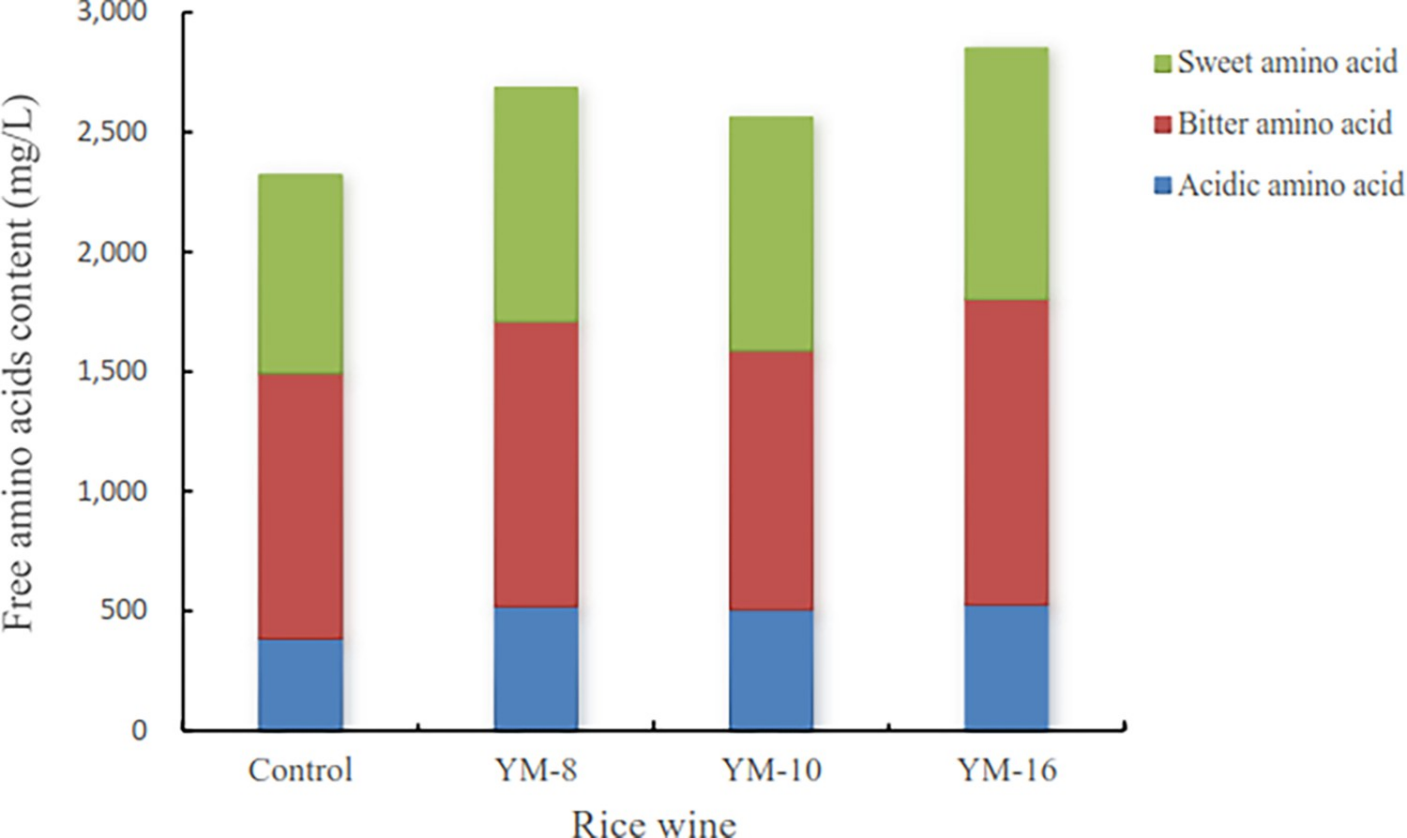
Free amino acid content of rice wine.

Amino acids produced by rice wine fermentation provide a nitrogen source for the yeast, which is beneficial to the generation and conversion of fusel alcohols, contributing to the aroma of rice wine. Free amino acids help to improve the nutritional value of the wine, enrich the flavor of the wine, and have a positive effect on the enhancement of the organoleptic flavor of rice wine; the most important amino acids in the formation of a special flavor of rice wine are sweet and bitter amino acids [48, 49]. As shown in Fig 4, the sweet amino acid content of the fermented rice wines of the screened strains varied greatly in alanine and lysine, with the sweet amino acid content of YM-16-fermented rice wine being significantly higher than that of the other three groups. The bitter amino acid content was slightly higher than that of the other three groups. Glutamic acid provided a strong acidic flavor, and the highest glutamic acid content was found in the fermented rice wine of YM-16, whereas the least glutamic acid was found in the fermented rice wine of YM-10, which was higher than that of the control group. These results are consistent with those of earlier research on the amino acid composition of rice wine during fermentation. Liang et al. [45] studied the dynamics of amino acids and microbial communities during the fermentation of Hong Qu glutinous rice wine and found that temperature had a significant effect on the production of bitter amino acids. They also reported that higher temperatures were associated with increased bitter, sweet, umami, and astringent amino acid contents, as well as total amino acid content. Moreover, they identified a positive correlation between the total bitter amino acid content and the relative abundance of certain microbial taxa, such as *Pediococcus*, *Saccharomyces*, *Lactobacillus*, *Monascus*, and *Halomonas*. Lu et al. [50] investigated the fermentation characteristics of traditional Dongshan old rice wine by studying the yeast strains, typical flavor components, and changes in ethyl carbamate content. They found that the histidine and arginine contents in rice wine were high, resulting in a higher bitter amino acid content, which decreased during aging.

In conclusion, this study highlights the importance of studying sweet and bitter amino acid contents in fermented rice wines made using different strains of mold. The findings suggest that YM-16 may be a promising strain for producing rice wine with high sweet amino acid content and unique flavor characteristics.

#### 3.5.2 Analysis of flavor components in fermented rice wine

**Fig 5:**
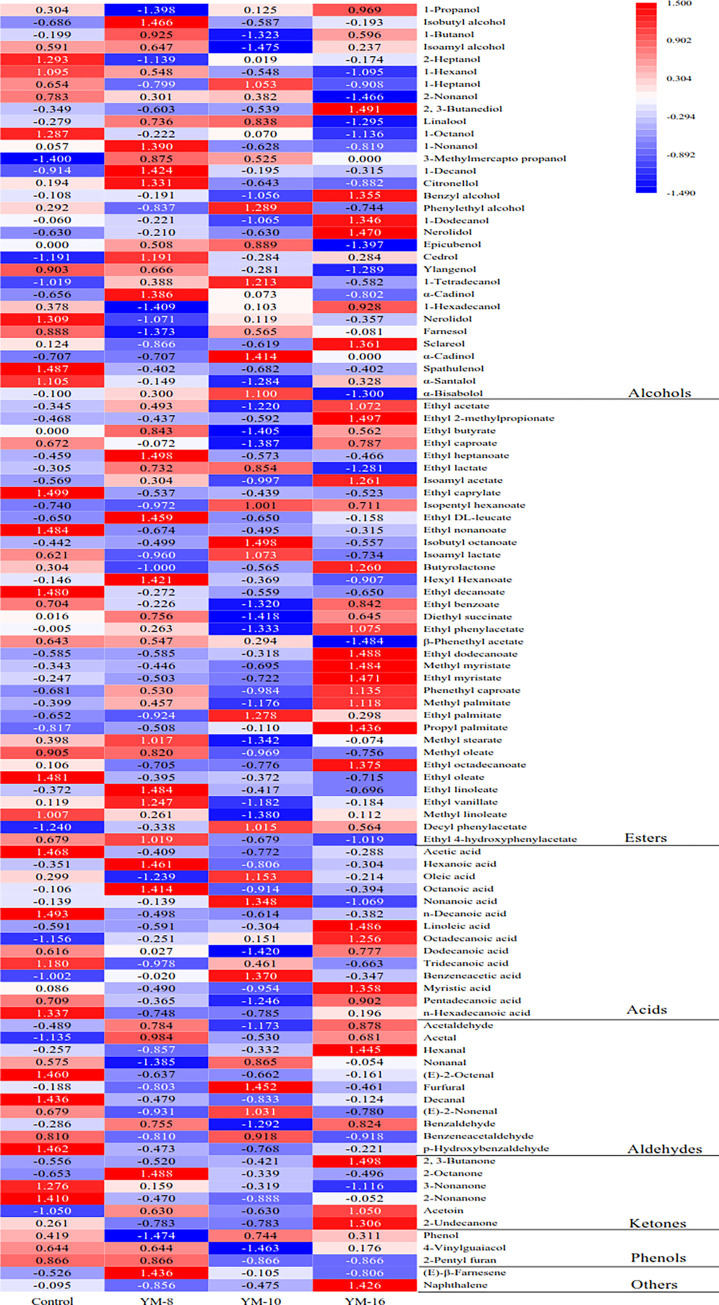
Heatmap of flavor components in fermented rice wine.

A comparison of flavor substances in rice wines fermented with different molds led to the identification of 104 flavor substances, including 32 alcohols, 36 esters, 14 acids, 11 aldehydes, six ketones, two phenols, and three other flavor components. A higher level of expression indicates that the content of flavor components in mold-fermented rice wine was higher and richer, as shown in Fig 5. The treatment groups showing flavor component expression levels greater than 1 were YM-16 (25 components), the control group (20 components), YM-8 (17 components), and YM-10 (14 components).

Higher alcohols are among the most important flavor substances in wine and are mainly derived from microbial fermentation and amino acid metabolism [50–53]. Among the alcohols with an expression of flavor substances greater than 1 in rice wine, there were six in YM-8, five in YM-10, five in YM-16, and six in the control group. The most abundant alcohols in rice wine were phenylethyl alcohol (sweet rose flavor), isobutyl alcohol (ethanol flavor), and isoamyl alcohol (banana flavor), which is consistent with the theory of key aroma components in rice wine [11, 52]. The results showed that the content of isobutyl alcohol was highest in the YM-8 treatment group, at 3.534±0.333 mg/L, followed by YM-16 at 2.818±0.159 mg/L and YM-10 at 2.648±0.157 mg/L.

The highest content of isoamyl alcohol in the fermentation treatment groups was in the YM-8 group, at 15.405±1.288 mg/L, followed by YM-16 at 13.704±1.336 mg/L and YM-10, showing a much lower content, at 6.594±0.221 mg/L. The content of phenylethyl alcohol was highest in the YM-10-fermented rice wine, at 15.189±0.517 mg/L, and lowest in the YM-8 group, at 10.658±0.586 mg/L. However, in general, each of the selected mold strains produced rice wine with characteristic alcohols at high enough content to yield the characteristic flavor of rice wine.

Lu et al. [50] examined the fermentation properties of traditional Dongshan old rice wine by analyzing the yeast strains, typical flavor compounds, and changes in ethyl carbamate content. They observed that the wine’s flavor compounds were primarily composed of alcohols and esters, such as phenethyl alcohol and diethyl succinate, with the ester content gradually increasing over time. Liu et al. [54] investigated the impact of mixed mold starters on the volatile flavor compounds in rice wine. They discovered that using a starter containing simultaneous inoculations of *R*. *chinonsis* R01, *A*. *niger* A20, *M*. *pusillus* M05, and *S*. *cerevisiae* S10 (RAM-S) led to differences in the aroma of rice wine, mainly due to variations in alcohol and ester amounts. Kim and Seo [55] explored the fermentation characteristics of rice wine produced from koji inoculated with *Aspergillus oryzae* KSS2 and *Rhizopus oryzae* KJJ39 isolated from a Korean fermentation starter. Their findings revealed that the alcohol content in rice wine samples fermented with wheat-bran koji was approximately 12% higher than that in the other samples.

These comparisons suggest that although there may be some variation in the exact concentrations of flavor substances among different studies, the key aroma components in rice wine, including phenylethyl alcohol, isobutyl alcohol, and isoamyl alcohol, remain consistent. Further research is needed to understand the factors influencing the variation in alcohol content and optimize the production process for the desired aroma profiles.

Esters are the most diverse flavor components of rice wine and are mainly produced by esterification reactions between higher alcohols and fatty acids. Esters are an important material basis for the special floral and fruity aromas in rice wine and can increase the complexity and typicality of the wine aroma [56, 57]. A number of esters with high expression (greater than 1) were found in the fermented rice wine produced in the present study: seven in YM-8, five in YM-10, twelve in YM-16, and five in the control group. Seven of the identified esters had an expression level greater than 1. The esters with the highest content in the rice wine were ethyl acetate (pineapple, apple flavor), ethyl caproate (sweet, fruit flavor), β-phenethyl acetate (flower and fruit flavor, peach flavor), and isoamyl acetate (sweet, fruit flavor), which can produce rice wine with special flower and fruit flavors [58, 59]. The contents of ethyl acetate, ethyl caproate, and isoamyl acetate in YM-16-fermented rice wine were the highest, with values of 3.095±0.188, 3.26±0.213, and 1.458±0.074 mg/L, respectively. The control group had the highest content of β-phenethyl acetate in fermented rice wine, which was 2.796±0.256 mg/L. Zheng et al. [60] studied the production of intensified Qu (IQ) and its effect on aroma variation during the fermentation of Huangjiu, a Chinese rice wine. They observed that adding pure yeast to Qu enhanced the fruit and floral aromas, and the content of esters, including ethyl acetate and isoamyl acetate, was noticeably higher in the IQ fermentation broth. These findings support the notion that specific microbial strains can significantly influence the aroma profiles of rice wines. Yan et al. [61] examined the effects of different *koji* types on rice wine aroma. Their analysis revealed that the amount and type of esters as well as the interactions between esters, alcohols, and acids play crucial roles in the sensory quality of rice wine. This study further confirms the significance of selecting appropriate *koji* strains to optimize the aroma composition of rice wine. In conclusion, the present study contributes to the understanding of the role of different fungal strains in ester production and the subsequent development of distinctive flavor profiles in fermented rice wine.

A number of acids were identified in the mold-fermented rice wine. Two in YM-8, three in YM-10, three in YM-16, and four in the control group—and these acids had an expression level greater than 1. If it has a fruity and floral aroma, it can enrich the flavor of rice wine. The highest content of octanoic acid in YM-8 was 0.913±0.075 mg/L, followed by the control group and YM-16, with values of 0.518±0.041 mg/L and 0.443±0.037 mg/L, respectively.

With respect to aldehydes, ketones, phenols, and other flavor components with high expression (greater than 1), there were two in YM-8, two in YM-10, five in YM-16, and five in the control group. Compared with esters and alcohols, the types and content of flavor components such as acids, aldehydes, and phenols in rice wine were significantly reduced, but they are still very important for the formation of whole rice wine flavor [4, 61].

**Fig 6:**
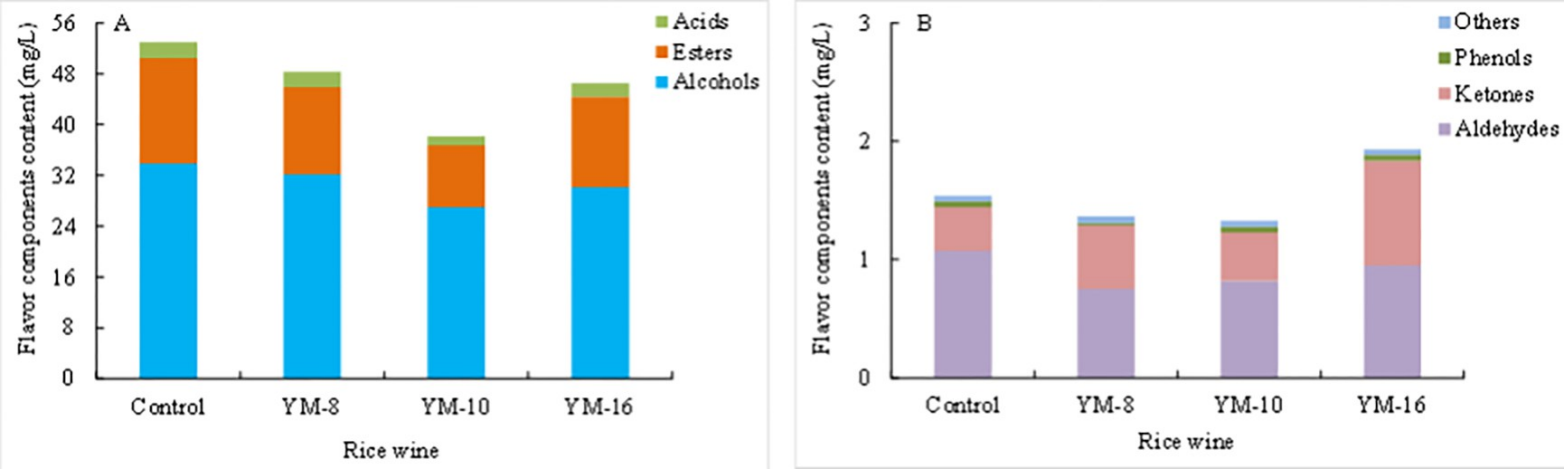
Flavor component content in rice wine.

The highest content of flavor substances was in the control group (54.498±1.968 mg/L), followed by YM-8 and YM-16 (49.727±2.439 mg/L and 48.486±2.684 mg/L, respectively) and YM-10 (39.534±1.000 mg/L), as shown in Fig 6. When the number of microorganisms in rice wine curds is complex, the interactions between microorganisms are favorable for the generation of flavor substances. Interactions are very favorable for the generation of flavor substances. The control group had a higher content, whereas YM-8 and YM-16 had lower contents of volatile aroma components than the control group and had the basic flavor substances needed for rice wine, which is conducive to the formation of rice wine aroma. YM-10 also had the flavor substances needed for rice wine, but its content was lower. The higher content of volatile flavors in the control group may be because the microorganisms in the control group are more diverse, which is conducive to the production of flavors. Xiang et al. [11] studied the use of *Mucor indicus* in coculture with *Rhizopus oryzae* to improve the flavor of Chinese turbid rice wine (CTRW). They found that co-culture altered the profiles of flavor compounds in CTRW, enhancing the concentrations of sweet amino acids, fruity and floral esters, and higher alcohols. This resulted in CTRW with a more intense aroma, harmonious taste, continuation, and full-body mouthfeel owing to the increased abundance of flavor compounds. The authors concluded that *Mucor indicus* is a promising species for co-culturing with *R*. *oryzae* to enhance the flavor of CTRW.

Ma et al. [62] investigated the correlation between brewing microorganisms and flavor compounds in different JIUQU rice wines from a macro perspective. Their study identified nine main characteristic flavor compounds in rice wine and 16 dominant microorganisms in rice wine starters. Strong correlations have been found between specific microorganisms and the formation of certain flavor compounds, such as *Ochrobactrum*, *Erwinia*, *Klebsiella*, and *Staphylococcus*, which affect the formation of ethyl isobutyrate, ethyl octanoate, ethyl butyrate, ethyl hexanoate, acetic acid, and linalool. *Monascus* showed a strong correlation with phenylethanol, linalool, and hexanal, indicating its contribution to flavor compound formation. In comparison to these studies, our research provides further evidence of the significance of aldehydes, ketones, and phenols on rice wine flavor, despite their relatively lower content compared to esters and alcohols. Our findings support the notion that specific microorganisms play crucial roles in the formation of distinct flavor compounds, as demonstrated by Zhao et al. [63].

#### 3.5.3 Correlation analysis of flavor substances and amino acids in fermented rice wine

**Fig 7:**
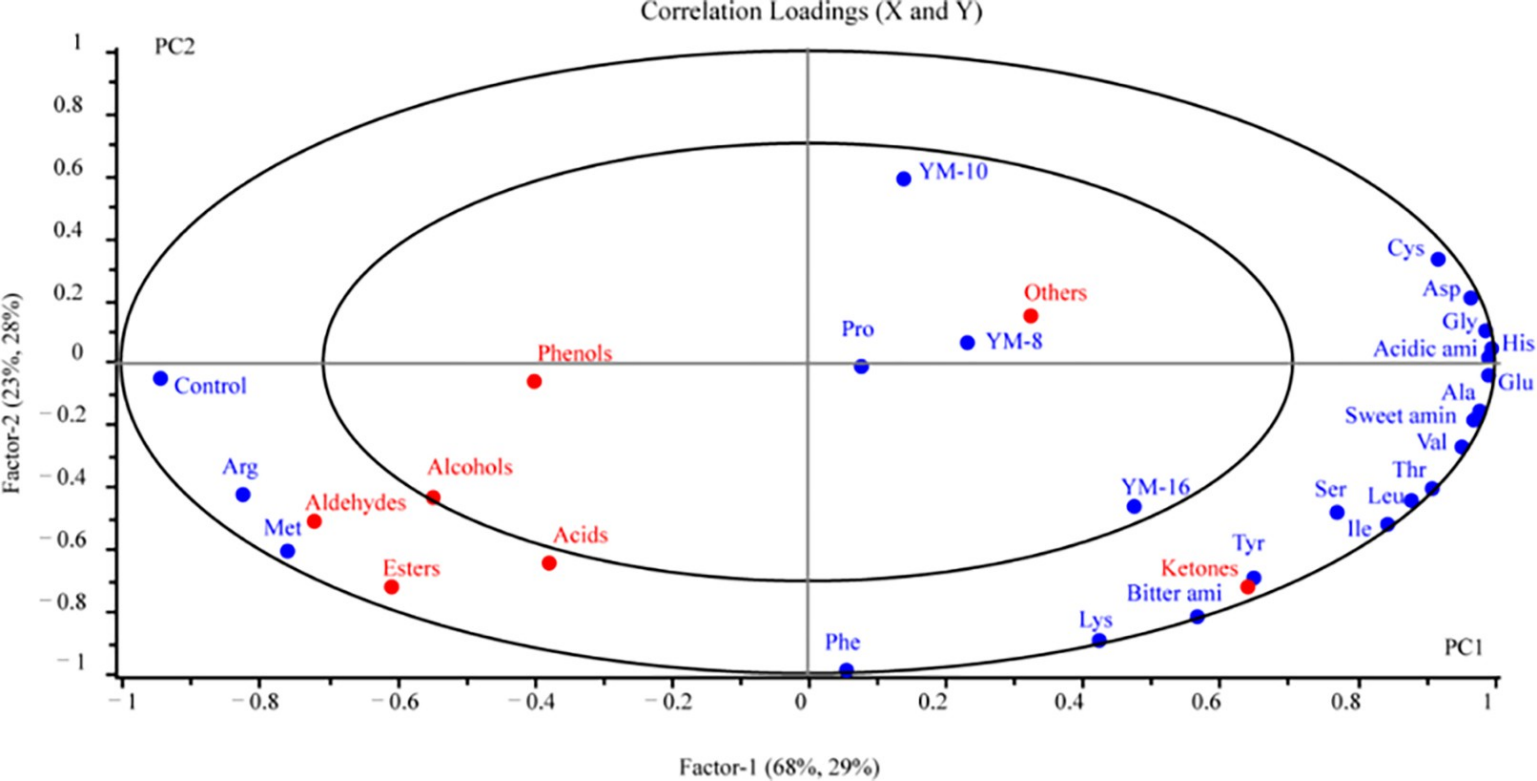
A brief overview of the PLSR correlation loading plot of rice wine for screened mold. The X-matrix was composed of 4 rice wine samples and amino acids, and the Y-matrix was composed of flavor substances.

PLSR was designed to investigate the connection between amino acids, flavoring agents, and rice wine produced by screened mold. The two enormous circles in the plot in Fig 7 show the 50% and 100% explained differences. The model’s R2(cum) for fitting to the independent variable was 0.981, Q2(cum) for prediction was 0.987, and RMSE was 0.001 or below, indicating that the model was stable and dependable. A model was created in our study utilizing four samples of rice wine (Control, YM-8, YM-10, and YM-16), amino acids as the X-matrix, and flavorings as the Y-matrix. Meanwhile, two significant PC1 and PC2 components that accounted for 97% of the cross-validated variance of the X-matrix and 51% of the cross-validated variance of the Y-matrix, respectively, were included in the developed PLSR model. As shown in Fig 7, the rice wine of the control was located on the left side, which was distinguishable from the YM-8, YM-10, and YM-16 rice wines positioned on the right side. Rice wine in the control group was significantly positively correlated with aldehydes, esters, Arg, and Met. In contrast, the ketones, others, and Profound in rice wine from the YM-8, YM-10, and YM-16 regions were positively correlated with acidic and sweet amino acids. This was similar to the flavor substances of rice wine in the control group. However, rice wine of the Control, YM-8, YM-10, and YM-16 groups did not significantly influence (*p* < 0.05) sensory features.

## 4. Conclusion

A comprehensive evaluation revealed that the YM-8, YM-10, and YM-16 molds are ideal strains for rice wine brewing. This selected mold, YM-16, has strong saccharification and fermentation ability, is a rich enzyme system, and improves the flavor of rice wine, thereby demonstrating its suitability as a production strain for brewing. In future rice wine production, molds with various advantageous characteristics can be combined to create rice wine with various microorganism fermentation flavors to cater to different preferences.

## Supporting information

S1 Fig (DOCX)

S2 Fig (XLSX)

S3 Fig (XLSX)

S4 Fig (XLSX)

S5 Fig (XLSX)

S6 Fig (XLSX)

S1 Table (XLSX)

S2 Table (XLSX)

S3 Table (XLSX)
