# Supplementary material for: Screening, identification, and characterization of molds for brewing rice wine: Scale-up production in a bioreactor
Source: PLoS One. 2024 Jul 2;19(7):e0300213. doi: 10.1371/journal.pone.0300213 (PMC11218956; doi:10.1371/journal.pone.0300213)
Supplement: S1 Fig — (DOCX) [file pone.0300213.s001.docx]

YM-8 ITS sequence：

ATTAACTAATGTATTGGCACTTTACTGGGATTTACTTCTCAGTATTGTTTGCTTCTATACTGTGAACCTCTGGCGATGAAGGTCGTAACTGACCTTCGGGAGAGACTCAGGACATATAGGCTATAATGGGTAGGCCTGTTCTGGGGTTTGATCGATGCCAATCAGGATTACCTTTCTTCCTTTGGGAAGGAAGGTGCCTGGTACCCTTTACCATATACCATGAATTCAGAATTGAAAGTATAATATAATAACAACTTTTAACAATGGATCTCTTGGTTCTCGCATCGATGAAGAACGTAGCAAAGTGCGATAACTAGTGTGAATTGCATATTCGTGAATCATCGAGTCTTTGAACGCAGCTTGCACTCTATGGATCTTCTATAGAGTACGCTTGCTTCAGTATCATAACCAACCCACACATAAAATTTATTTTATGTGGTGATGGACAAGCTCGGTTAAATTTAATTATTATACCGATTGTCTAAAATACAGCCTCTTTGTAATTTTCATTAAATTACGAACTACCTAGCCATCGTGCTTTTTTGGTCCAACCAAAAAACATATAATCTAGGGGTTCTGCTAGCCAGCAGATATTTTAATGATCTTTAACTATGATCTGAAGTCAAGTGGGACTACCCGCTGAACTTAA

YM-10 ITS sequence：

GAAGGATCATTAATTATGTTAAAGCGCCTTACCTTAGGGTTTCCTCTGGGGTAAGTGATTGCTTCTACACTGTGAAAATTTGGCTGAGAGACTCAGACTGGTCATGGGTAGACCTATCTGGGGTTTGATCGATGCCACTCCTGGTTTCAGGAGTACCCTTCATAATAAACCTAGAAATTCAGTATTATAAAGTTTAATAAAAAACAACTTTTAACAATGGATCTCTTGGTTCTCGCATCGATGAAGAACGTAGCAAAGTGCGATAACTAGTGTGAATTGCATATTCAGTGAATCATCGAGTCTTTGAACGCAGCTTGCACTCTATGGTTTTTCTATAGAGTACGCCTGCTTCAGTATCATCACAAACCCACACATAACATTTGTTTATGTGGTGATGGGTCGCATCGCTGTTTTATTACAGTGAGCACCTAAAATGTGTGTGATTTTCTGTCTGGCTTGCTAGGCAGGAATATTACGCTGGTCTCAGGATCTTTTTTTTTGGTTCGCCCAGGAAGTAAAGTACAAGAGTATAATCCAGTAACTTTCAAACTATGATCTGAAGTCAGGTGGGATTACCCGCTGAACTTAAGC

YM-16 ITS sequence：

ACGGGAACGTGGGTTCTAGCGAGCCCACCTCCCACCCGTGTTTACTGTACCTTAGTTGCTTCGGCGGGCCCGCCATTCATGGCCGCCGGGGGCTCTCAGCCCCGGGCCCGCGCCCGCCGGAGACACCACGAACTCTGTCTGATCTAGTGAAGTCTGAGTTGATTGTATCGCAATCAGTTAAAACTTTCAACAATGGATCTCTTGGTTCCGGCATCGATGAAGAACGCAGCGAAATGCGATAACTAGTGTGAATTGCAGAATTCCGTGAATCATCGAGTCTTTGAACGCACATTGCGCCCCCTGGTATTCCGGGGGGCATGCCTGTCCGAGCGTCATTGCTGCCCATCAAGCACGGCTTGTGTGTTGGGTCGTCGTCCCCTCTCCGGGGGGGACGGGCCCCAAAGGCAGCGGCGGCACCGCGTCCGATCCTCGAGCGTATGGGGCTTTGTCACCCGCTCTGTAGGCCCGGCCGGCGCTTGCCGAACGCAAATCAATCTTTTTCCAGGTTGACCTCGGATCAGGTAGGGATACCCGCTGAACTTAAGCATATCAATAAGCGGAGGAA
